# The Enduring Hypoxic Response of *Mycobacterium tuberculosis*

**DOI:** 10.1371/journal.pone.0001502

**Published:** 2008-01-30

**Authors:** Tige R. Rustad, Maria I. Harrell, Reiling Liao, David R. Sherman

**Affiliations:** 1 Seattle Biomedical Research Institute, Seattle, Washington, United States of America; 2 Department of Pathobiology, University of Washington, Seattle, Washington, United States of America; 3 Fred Hutchinson Cancer Research Center, Seattle, Washington, United States of America; Wellcome Trust Sanger Institute, United Kingdom

## Abstract

**Background:**

A significant body of evidence accumulated over the last century suggests a link between hypoxic microenvironments within the infected host and the latent phase of tuberculosis. Studies to test this correlation have identified the *M. tuberculosis* initial hypoxic response, controlled by the two-component response regulator DosR. The initial hypoxic response is completely blocked in a *dosR* deletion mutant.

**Methodology/Principal Findings:**

We show here that a *dosR* deletion mutant enters bacteriostasis in response to *in vitro* hypoxia with only a relatively mild decrease in viability. In the murine infection model, the phenotype of the mutant was indistinguishable from that of the parent strain. These results suggested that additional genes may be essential for entry into and maintenance of bacteriostasis. Detailed microarray analysis of oxygen starved cultures revealed that DosR regulon induction is transient, with induction of nearly half the genes returning to baseline within 24 hours. In addition, a larger, sustained wave of gene expression follows the DosR-mediated initial hypoxic response. This Enduring Hypoxic Response (EHR) consists of 230 genes significantly induced at four and seven days of hypoxia but not at initial time points. These genes include a surprising number of transcriptional regulators that could control the program of bacteriostasis. We found that the EHR is independent of the DosR-mediated initial hypoxic response, as EHR expression is virtually unaltered in the *dosR* mutant.

**Conclusions/Significance:**

Our results suggest a reassessment of the role of DosR and the initial hypoxic response in MTB physiology. Instead of a primary role in survival of hypoxia induced bacteriostasis, DosR may regulate a response that is largely optional *in vitro* and in mouse infections. Analysis of the EHR should help elucidate the key regulatory factors and enzymatic machinery exploited by *M. tuberculosis* for long-term bacteriostasis in the face of oxygen deprivation.

## Introduction

*Mycobacterium tuberculosis* (MTB) infections can persist without symptoms for decades before reactivation [1], [2], facilitating dissemination to distant locations and new, naïve hosts. This adaptation plays a key role in enabling a slow-growing, non-motile bacterium without a significant animal reservoir to spread across the globe and achieve its remarkable level of prevalence. Up to a third of all people are skin test positive for MTB infection [3], [4]. In addition, factors that promote TB latency may also be important during active TB disease. MTB in humans can be metabolically heterogeneous, with active and quiescent lesions adjacent to one another [5], [6]. Difficulty in eradicating bacilli from quiescent lesions may underlie the extended chemotherapeutic regimens needed to treat active TB. Length of treatment in turn fuels patient non-compliance and development of drug resistant strains [7]. Understanding the mechanisms used by MTB to enter into, survive, and reactivate from latent disease states is critical given the global burden of tuberculosis and the dwindling number of effective TB treatments to combat the emergence of multi-drug resistant (MDR) and extensively drug resistant (XDR) strains.

Granuloma formation is the hallmark of TB infection. Granulomas are formed by activated macrophages and other host components that surround infected lung tissue, isolating the infected cells in an organized structure and creating an environment that suppresses MTB replication [2], [8]–[14]. Granulomas are thought to limit bacterial growth in a variety of ways including oxygen and nutrient deprivation, acidic pH, and production of host factors such as nitric oxide. Of these, hypoxia is the best-studied, with much work focused on *in vitro* models of hypoxia-induced dormancy. Tuberculosis bacilli exposed to hypoxia *in vitro* cease replicating but can remain viable and virulent for years [15]. These nonreplicating bacilli have a drug susceptibility profile resembling that of latent TB infections [16]–[20]. Further studies are needed to validate the hypoxic models of latency and identify mechanisms used by MTB to enter into, persist in, and exit from latent disease states.

The initial response of MTB to hypoxia is tightly regulated by the two-component response regulator DosR (also called DevR, Rv3133c) [21]–[23]. Phosphorylation of DosR by either of two sensor histidine kinases, DosS or DosT, leads to induction of a set of ∼50 genes [24], many of unknown function. A consensus DosR binding sequence has been identified in the upstream regions of many genes from the DosR regulon [22], [25]. The DosR regulon is also induced in response to nitric oxide, in standing culture (which generates a hypoxia gradient), and following infection of macrophages, mice, and guinea pigs [26]–[28]. Some of these conditions are marked by significant bacterial replication, suggesting that the role of DosR may not be specific to latency and that other factors may be involved in the MTB latency response.

The studies described here characterize the MTB response to hypoxia in more depth. We show that the initial hypoxic response regulated by DosR contributes modestly to survival under hypoxic conditions *in vitro* but is dispensable for virulence in mice. Further transcriptional analysis under hypoxic conditions *in vitro* revealed that induction of the DosR regulon is transient, with expression of nearly half of the genes returning to baseline by 24 hours. However, we noted a significant additional transcriptional response. Comprised of over two hundred genes that remain induced for days, this Enduring Hypoxic Response (EHR) is both more extensive and more stable than the DosR response. Analysis of the transcriptional profile of the DosR mutant over a hypoxic time-course showed that the EHR is largely independent of the DosR regulon. Additional study of the EHR may provide important clues to MTB mechanisms of survival during bacteriostasis.

## Results

### *In vitro* and *in vivo* phenotypes of the dosR mutant

The initial MTB response to hypoxia is regulated by DosR, and deletion of either the regulator or both sensor kinases that form the two-component response system leads to disregulation of the response [21], [22], [24], [29]. This tight regulation in response to hypoxic stimuli suggested a potential role for the DosR regulon in responding to hypoxic stress. To explore this hypothesis, we exposed a *dosR* deletion mutant of MTB to several models of *in vitro* hypoxia to measure the survival of the mutant relative to wild-type.

**Figure 1:**
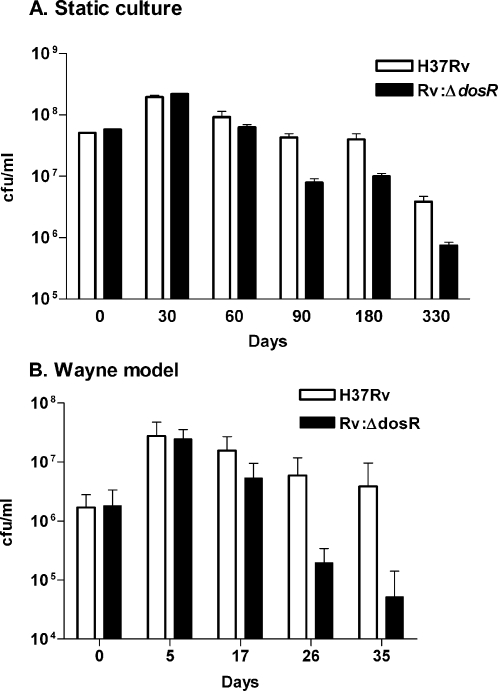
Survival of H37Rv and H37Rv:Δ*dosR* in hypoxia-induced bacteriostasis *in vitro*. Colony forming units per ml of parent strain H37Rv (white bar) and H37Rv:Δ*dosR* (black bar) were counted at each time point in the (A) standing culture and (B) Wayne models of hypoxia. Data are from one representative experiment of 3 (standing culture) or 5 (Wayne model). Each bar represents a minimum of 3 biological replicates.

In the defined hypoxic model, a constant flow of low oxygen gas over the surface of a stirred, early log phase culture is used to deplete the oxygen in a rapid and highly reproducible way. This model was used initially to characterize the MTB transcriptional response to hypoxia; the DosR regulon is induced within two hours and bacteriostasis is evident within 24 hours, with less than a single doubling occurring after the initial exposure to low oxygen conditions [21]. In this system, the *dosR* mutant and wild-type strains showed no survival difference over a one-week period (Figure S1). Longer time points are not feasible in this system, due to complications from evaporation. To test survival following exposure to prolonged hypoxia, we employed a standing culture model. Wild-type and mutant bacilli were cultured in competition in small cryovials with no head space for up to 1 year. By 90 days, survival of the *dosR* mutant was about one log lower than that of wild-type (Figure 1a). This difference was still evident after one year in standing culture.

The most frequently used experimental approach to hypoxia-induced MTB dormancy is the defined headspace model of non-replicating persistence (NRP) described by Lawrence Wayne and colleagues [17], [30], [31]. In this model MTB is grown in stirred, air-tight tubes with a defined headspace-to-culture ratio. The oxygen in the tube is depleted gradually over the course of days by the growing bacilli, with induction of the DosR regulon seen as early as 5 days [27]. At 17 days, well after the DosR regulon is induced [27] and bacteriostasis is firmly established, the *dosR* mutant showed a modest survival defect (Figure 1b). The drop in relative viability increased to nearly fifty fold after 26 days, and after 35 days in the Wayne model survival of the mutant was ∼75-fold less than the wild-type strain. This result is consistent with the ∼2 log drop shown earlier with a related MTB mutant in which DosR expression is disrupted [26], though significantly less than the 1000-fold drop in viability reported in a *dosR* deletion in a *Mycobacterium bovis* BCG vaccine strain [32].

**Figure 2:**
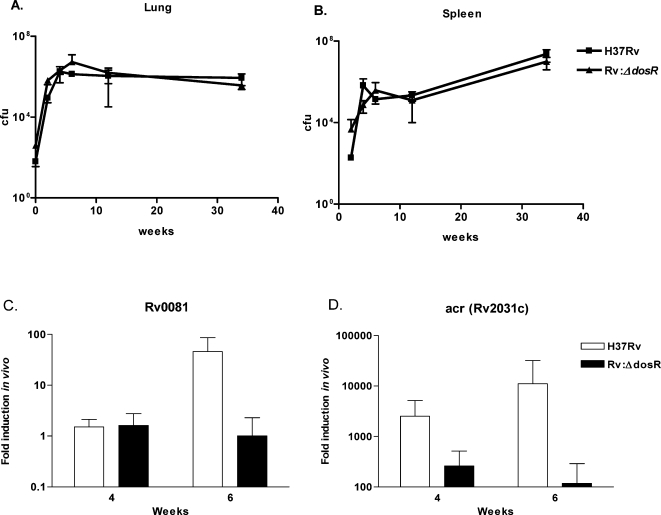
The phenotype of H37Rv:Δ*dosR* in mouse model is indistinguishable from wild type. Bacterial burdens of H37Rv (squares) and H37Rv:Δ*dosR* (triangles) in the (A) lungs and (B) spleens of C57BL/6 mice over the course of infection. Each point represents the average of three experiments with 3–5 animals per time point in each experiment. Error bars represent standard deviation. (C&D) Disregulation of the DosR regulon in a *dosR* mutant during murine infection was verified by quantitative real-time PCR of sentinel genes Rv0081 (C) and Rv2031c (D) from H37Rv (white) and H37Rv:Δ*dosR* (black) mRNA isolated from mouse lungs at 4 and 6 weeks post infection. Values were normalized to housekeeping gene SigA and shown as the ratio of normalized *in vivo* expression to log phase *in vitro* levels of expression.

To assess the link between DosR regulon expression and virulence or persistence *in vivo*, the *dosR* mutant was used to infect C57BL/6 mice. The bacterial burden as measured by colony forming units (cfu) (Figure 2a) and histopathology (data not shown) of the mutant was indistinguishable from the parent strain H37Rv. Additional experiments with more susceptible DBA2 and C3He/FEJ mice confirmed that DosR is dispensable for persistence and virulence in these models (Figures S2 and S3). To test if the DosR regulon might be expressed *in vivo* in the absence of *dosR* by an alternate regulatory pathway, expression of two sentinel genes from the DosR regulon in wild-type and mutant-infected mice were monitored by quantitative real-time PCR over the course of infection (Fig 2b). In each case the expression of the DosR regulon gene was markedly lower in the DosR mutant, confirming the disregulation of the regulon in the mutant strain.

### Transcriptional analysis of a hypoxic time course

Non-replicating persistence *in vitro* and chronic infection *in vivo* occur despite disruption of the DosR regulon, suggesting that additional genes must contribute to these phenotypes. The initial hypoxic response controlled by DosR was defined as those MTB genes induced following two hours of exposure to hypoxia in the defined hypoxic model [21], [22]. To characterize MTB responses to hypoxia downstream of this response we followed the transcriptional profile of H37Rv through an extended time course, with samples taken from aerated log phase cultures and cultures exposed to four, eight, twelve, 24, 96, and 168 hours of hypoxia. Each hypoxic time point was analyzed with at least three biological replicates using high density oligonucleotide microarrays with a minimum of four on-chip replicates (NCBI/GEO accession number GSE9331).

After the initial hypoxic response, expression of the DosR regulon wanes. The ∼50 genes of the regulon are maximally induced early in the time course and gradually decline ([21] and Table S1). By 24 hours, about half the regulon is no longer significantly induced. The twenty six genes that remain induced were initially the most powerful responders to DosR. They remain induced throughout the time course, though at levels well below their initial maximum.

### The Enduring Hypoxic Response (EHR)

**Figure 3:**
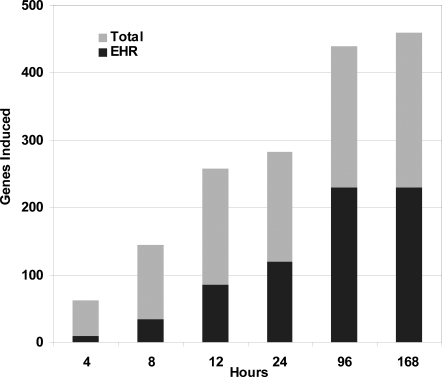
Enduring hypoxic response (EHR) dominates the hypoxic time course. Numbers of genes significantly induced over log phase (>two fold, FDR<0.4%) were tabulated at each time point over a hypoxic time course. Each bar represents the total number of genes (gray) and genes from the EHR (black).

A second transcriptional response, much larger than the DosR regulon and induced for a much longer period, is evident in a plot of the number of genes significantly induced in response to hypoxia (>2 fold, false discovery rate <0.4%, Figure 3 total bar). The number of induced genes increased until 96 hours into the experiment. The growth of this gene set was largely additive- each subsequent set contained most of the genes induced at the preceding time point. However by four days the number of induced genes seemed to stabilize around an Enduring Hypoxic Response (EHR), which we define as the set of MTB genes not induced initially that are significantly up-regulated at four and seven days of hypoxia. A set of 230 genes meet these criteria. (Figure 3, Table S2). There are genes specifically induced at each time point, but from 4 days on the EHR comprises the majority and the most highly expressed of the genes induced. As early as one day of hypoxia 126 of the EHR genes are already induced.

The EHR was compared to previously published microarray-generated gene lists and the number of overlapping genes was tabulated (Table 1). This comparison was standardized for lists of varying sizes based on the size of the EHR in relation to the genome to generate an estimate of the number of genes expected to be shared between any two gene lists by random chance. The EHR shows significant overlap with other array studies of non-replicating persistence. The overlap with microarray analyses of the Wayne model previously reported by Voskuil et al. is several times that expected by random chance [27]. As previously reported, the DosR regulon also overlaps significantly with the Wayne model array results presented by Voskuil et al.

**Table 1:** Comparison of EHR to previously published array analysis of bacteriostatic models and control comparisons.

	Condition	Total	EHR: 230 Genes		DosR: 49 genes	
			Overlap (By Chance)		Overlap (By Chance)	
**Starvation ** a	4 hours ND	170	**23**	(10)	**0**	(2)
	1 day ND	250	**54**	(15)	**0**	(3)
	4 day ND	276	**47**	(16)	**0**	(3)
**Wayne model ** b	NRP 4 days	78	**8**	(5)	**42**	(1)
	NRP 20 days	177	**73**	(10)	**33**	(2)
	NRP 80 days	9	**5**	(1)	**0**	(0)
**Control stresses ** c	pH 4.8	195	**18**	(11)	**1**	(2)
	Rifampin	375	**15**	(22)	**2**	(5)
	H2O2	199	**6**	(12)	**3**	(2)

a Starvation data from Betts et al [33]

b Wayne model data from Voskuil et al [27]

c Control stress data from Boshoff et al [35]

Another stimulus associated with non-replicating persistence *in vitro* is nutrient deprivation. Two groups have recently described the transcriptional profile of nutrient deprived non-replicating MTB [33], [34]. Interestingly, there is considerable overlap with the EHR and the set of genes induced in the nutrient deprivation model of MTB latency, again well beyond the overlap expected by random chance. In contrast, there is no overlap between genes induced by nutrient deprivation and the DosR regulon. As a control, we measured the overlap of the EHR with array profiles of MTB cultures in the presence of 2 non-similar stresses and found the overlap near or below that expected by chance [35].

A survey of the annotated functional categories [36] of EHR genes reveals that a dominant feature of this gene list is the large percentage of putative regulatory proteins, more than double the number expected by chance (Table 2). These regulatory proteins include the iron uptake regulators FurA and FurB, three members of the WhiB gene family, and the two-component response sensor PhoP (the other component of that system falls just below the significance threshold). The alternative sigma factors SigE and SigH are also induced during the EHR.

**Table 2:** Functional categories of the EHR.

Functional categories	EHR #genes	%EHR	%genome
conserved hypotheticals/unknown	80	34.8	31.9
intermediary metabolism and respiration	54	23.5	22.4
cell wall and cell processes	22	9.6	18.8
lipid metabolism	7	3.0	5.9
information pathways	6	2.6	5.8
regulatory proteins	30	13.0	4.8
PE/PPE	13	5.7	4.2
insertion seqs and phages	6	2.6	3.7
virulence, detoxification, adaptation	12	5.2	2.6

Several other genes and categories of genes represented in the EHR are of note. Not surprisingly, given the aerobic conditions under which gene function is typically assessed, the EHR includes a high proportion of genes of unknown function. There are also more genes than expected by chance annotated as involved in virulence, detoxification, and adaptation, including a number of heat shock proteins. RelA, regulator of the stringent response, is induced as part of the EHR [37]. This response has been implicated in the survival of MTB under nutrient deprivation and anaerobic growth conditions. Genes annotated as oxidoreductases and cytochromes are also prominent in the EHR, and these genes are likely involved in anaerobic metabolism. There are also several induced genes putatively involved in sulfur metabolism. These include the atypical sulfur transporter *sse1*, the APS synthase complex *cysD/cysN*, the methionine synthase gene *cysM*, and several potentially unrelated genes involved in synthesis of sulfur-containing compounds such as thioredoxin and ferrodoxin.

### The EHR and the DosR regulon

**Figure 4:**
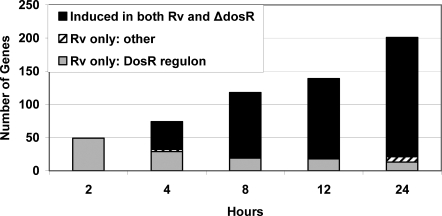
Downstream hypoxic response independent of DosR regulon. Each bar represents the number of genes induced in H37Rv at each point during a short hypoxic time course (>two fold in all three arrays). The DosR regulon represents a dwindling fraction of the genes induced over time (gray). Very few genes were induced in H37Rv and not in the *dosR* mutant (diagonal lines). The overwhelming majority of genes induced in the parent strain at later hypoxic time points were also induced in the mutant (black).

We sought to define the link between the initial hypoxic response controlled by DosR and the EHR. Accordingly, three paired H37Rv and H37Rv:*ΔdosR* hypoxic time courses were conducted. Samples were taken at four, eight, twelve, and 24 hours of exposure to hypoxia. As before, microarrays were used to compare RNA from each strain at each hypoxic time point to log phase expression. To avoid threshold effects caused when modestly induced genes wobble across the two-fold threshold, we applied a more stringent criterion (>2 fold in all three arrays) to define the set of genes induced at each time-point. The genes induced at each time-point were divided into three classes: (1) induced in both H37Rv and the *dosR* mutant, (2) the DosR regulon genes, which are not induced in the *dosR* mutant, and (3) the genes outside the DosR regulon that are induced in H37Rv but not the mutant (Figure 4). If EHR expression depends significantly on the DosR response, we would expect a large number of genes in that final category.

Surprisingly, almost none of the nearly 200 genes induced by 24 hours (not including DosR regulon genes) appear to be DosR-dependent. At each step in the time course the set of genes induced increased, and these new genes were induced both in the wild type and the mutant. As expected, the DosR regulon is not induced in the *ΔdosR* mutant, and these constitute the majority of hypoxia induced genes that are disregulated. The EHR constitutes nearly half of the genes induced at one day and those genes remain induced throughout the time course.

## Discussion

The DosR-regulated response to hypoxia has been suggested to play a major role in mycobacterial dormancy, as is apparent from the name DosR, Dormancy survival Regulator. However, the data presented here suggest a more modest role for DosR in survival of hypoxia-mediated bacteriostasis. *In vitro*, MTB lacking DosR show no survival defect in the defined hypoxic model and only a modest effect in the standing culture hypoxic model. However MTB are somewhat impaired for survival in the Wayne model. Wayne model bacilli are likely to experience stresses in addition to hypoxia, such as nutrient depletion and toxic waste accumulation.

In the Wayne model oxygen is slowly depleted as it is consumed, allowing the bacilli to adapt to hypoxia in a gradual manner. Previous work suggested that rapid depletion of oxygen resulted in significant bacterial killing of log phase cultures, with less than 0.2% of cells surviving 10 days of hypoxia [31]. In contrast, we have found that the rapid depletion of oxygen does not result in a massive drop in viability. The difference may be due to the methods used to achieve hypoxia. Whatever the case, the defined hypoxic model is a highly reproducible alternative to the Wayne model.

In the Wayne model, neither the modest survival defect of the DosR mutant nor the cessation of replication aligns with the timing of DosR regulon induction [27]. Perhaps DosR regulon induction helps prime MTB for long term bacteriostasis by sequestering nutrients and triggering changes that, once complete, no longer require regulon expression. For example triacylglycerol-containing lipid bodies may serve as energy reserves for MTB [38], thus the induction of *tgs1* by DosR may pave the way for a downshift in the bacilli's metabolism. Of course, DosR may contribute to MTB physiology in ways that are not yet appreciated.

The DosR regulon is strongly induced when MTB infects mice, but we found that deletion of DosR did not compromise bacterial survival or virulence. QRT-PCR of sentinel genes confirmed that DosR regulon genes are indeed disregulated when *dosR*-mutant MTB infect mice. A previous report also showed that the *dosR* mutant is not attenuated in mice; in fact, these authors observed modest hypervirulence [39]. In contrast, *dosR*-mutant strains may be slightly less virulent in guinea pigs [29]. These differences could reflect differences in TB lesions in these systems. TB granulomas in mice are not especially hypoxic [40], [41]. Alternatively, slightly different results for DosR mutants *in vivo* may result from subtle variations in experimental methods that emphasize uncharacterized functions of the DosR regulon. However, our *in vivo* results, with the *dosR* mutant phenotype indistinguishable from wild-type, were consistent across three different mouse strains and two different routes of infection (data not shown).

Rarely in the host does MTB have access to the high levels of aeration found in rolling or stirred *in vitro* cultures. Although the DosR regulon is off in rolling aerobic cultures, very small drops in oxygen tension lead to rapid induction of the regulon. During the early stages of infection, bacilli are primarily found in macrophages within lung tissue. Under these conditions, oxygen tension is likely reduced relative to rolling cultures *in vitro*, however bacilli are replicating and DosR is induced [42]–[45]. In fact, in the natural history of TB infections the only conditions in which oxygen tension is likely high enough to mimic rolling culture conditions (where DosR is not induced) are in caseous lesions that open into airways and during aerosol transmission to a new host. Based on data presented here, we would also predict that DosR regulon expression would be reduced in lesions that remain hypoxic for extended periods of time. Thus, the DosR regulon is expressed *in vivo* as the bacilli are increasing in number. This expression pattern suggests that the DosR regulon's role is not simply as a latency trigger but functions at all times during the early stages of infection.

The minimal phenotypic effect of DosR deletion highlighted the question of which genes and mechanisms are used by MTB to enter and maintain bacteriostasis triggered by hypoxia. The response subsequent to the initial hypoxic response mediated by DosR contains a larger number of highly induced genes that remain induced for days after replication stops. This hypoxic response is dominated by a core of stably induced genes we name here the Enduring Hypoxic Response (EHR), and is observed in both wild-type and *dosR* mutant strains. We are currently dissecting this response to better understand the ways in which MTB enters into and survives bacteriostasis.

Analysis of the genes of the EHR should offer insights into the hypoxic response of MTB and the resulting bacteriostasis. Not surprisingly, there is considerable overlap with genes induced in the defined hypoxia model and the previously reported Wayne model of hypoxia. Moreover, the fraction of genes induced in the Wayne model that are also part of the EHR increases over the hypoxic time course. We also observe that, unlike the DosR regulon, the EHR shows a substantial overlap with the MTB genes induced by nutrient deprivation. Exploring common themes among the various *in vitro* models may help identify genes and processes essential for bacteriostasis in general, rather than any specific condition used to trigger replication arrest.

The genes repressed during the defined hypoxic time course are primarily well characterized genes involved in normal aerobic growth. Once the shift is made to anaerobiosis, energy generation and the regeneration of NAD^+^ become significant challenges. Surprisingly, many of the genes predicted to be involved these processes are not induced or are even repressed. These include the oxygen-independent NADH dehydrogenase complex (*ndh, ndhA, sdhA, B, C, D*), the nitrate (*narG, H, I, J*) and nitrite (*nirA, B,D*) reductase complexes, lactate dehydrogenase (*lld1, lld2*), glycine dehydrogenase (*gcvB*), and the isocitrate lyase gene of the glyoxylate shunt (*icl*). This is surprising given that active transcription and metabolism is occurring in these nonreplicating cells, as evidenced by the production and maintenance of mRNA. This poses an intriguing dilemma- what is the metabolic pathway used to generate energy in hypoxic MTB?

We were surprised to find that blocking expression of the DosR regulon had very little effect on subsequent expression of the EHR. The genes disregulated at later time points are almost all members of the DosR regulon that remain induced in wild type and fail to be induced in the mutant. This result provides further evidence that the DosR regulon is not required for entry or survival of bacteriostasis.

The EHR may contain the machinery used to enter into and survive latency. We hope to identify key components of this process that we can target for disruption, a challenging endeavor given the size of the EHR and the large number of regulatory proteins that it includes. Preliminary experiments have shown that the EHR is resilient in response to disruption of some of those regulatory factors. We are currently analyzing mutants in several putative EHR transcription factors for their transcriptional pattern and phenotype in the defined hypoxic model. We expect that further analysis of the *dosR* regulon and EHR will elucidate common properties and shared mechanisms with other dormancy models. These insights into the hidden life of dormant MTB will aid efforts to identify drug targets for latent infection.

## Materials and Methods

### Strains, culture conditions, and hypoxic models

Experiments were performed using H37Rv (ATCC 27294) or the H37RvΔ*dosR::kan* mutant [21], [22] grown at 37°C in Middlebrook 7H9 supplemented with ADC and 0.05% Tween (Beckton Dickinson) in rolling culture. Working stocks were expanded from frozen aliquots shortly before experiments began. The defined hypoxic model was performed as previously described [21]. Briefly, a 200 ml culture was grown to mid-log phase (A600 =  0.3–0.5), diluted in media to a starting A600 of 0.1, and 500 ml of the diluted culture was transferred to a 1 L three-armed spinner flask (Corning). Cultures were constantly stirred at 60 RPM for the duration of the experiment. Low oxygen gas (0.2% O_2_ with N_2_ balance) was constantly flowed over the culture at 0.15 sq. ft/min. through one arm. Another arm was used as a sampling port. Aliquots were removed at each time point, pelleted at 2000 g for 5 minutes, frozen on dry ice and stored at −80° until processed for RNA.

The Wayne model was performed as previously described [16], [17]. Occasionally, replicates gained significant turbidity, suggesting aerobic contamination of the hypoxic environment, and were excluded from downstream analysis. To enhance reproducibility, wild-type (kan-S) and mutant (kan-R) strains were co-incubated in individual tubes. Proportions of each strain were determined by plating both with and without kanamycin selection. Oxygen-limited stationary cultures were grown in 2 ml cryovials (Sarstedt) filled to the rim with bacteria at a starting A600 of 0.05 in 7H9 media. Tubes were tightly sealed, wrapped with parafilm, and incubated at 37°C. At each time point, three vials of each strain were vortexed, diluted in 7H9 broth, and plated for colony forming units (cfu) on 7H10 plates.

### *In vivo* experiments

All mouse experiments were performed according to established protocols approved by the University of Washington Institutional Animal Care and Use Committee. Animal experiments were performed in 6–8 week old mice (Jackson Laboratories) of three different strains: C57BL/6, DBA/2J and C3He/FeJ. Mice were maintained in a biosafety level 3 specific pathogen-free animal facility. Aerosol infections were performed as previously described [46]. Frozen bacterial stocks were thawed, sonicated, diluted to ∼10^6^ bacteria/ml, and nebulized in an aerosol infection chamber (Glascol) containing the mice. Mice were sacrificed at appropriate time points and the lungs were removed. The lower right lobe was homogenized in PBS/0.05% Tween 20 and plated as serial dilutions on 7H11 Selective Agar (Remel). Colonies were counted after 2–3 weeks of incubation at 37°C. The infectious dose was determined by plating whole-lung homogenates from 5 mice in each group on day 1.

### RNA extraction

RNA was extracted from cell pellets as previously described [21]. Pellets were resuspended in Trizol, transferred to a tube containing Lysing Matrix B (QBiogene, Inc.), and vigorously shaken at max speed for 30 sec in a FastPrep 120 homogenizer (Qbiogene) three times, with cooling on ice between steps. This mixture was centrifuged at max speed for 1 min and the supernatant was transferred to a tube containing 300 µL chloroform and Heavy Phase Lock Gel (Eppendorf North America, Inc.), inverted for one minute, and centrifuged at max speed. The aqueous phase was then precipitated with 270 µL isopropanol and 270 µL high salt solution (0.8M Na citrate, 1.2M NaCl). RNA was cleaned using an RNeasy kit following manufacturer's recommendations (Qiagen).

### Quantitative Real-Time PCR

cDNA was generated using 0.5–1 µg of total RNA with Thermoscript reverse trancriptase (Invitrogen) according to manufacturer's specification using specific primers. Mock reactions with no RT were done on each sample to assay for DNA contamination. Two-step real time PCR was performed as previously described by Dolganov and colleagues [47]. Primary amplification was performed with Platinum Taq polymerase (Invitrogen). Each PCR contained 2 µl of cDNA material from the RT step and specific primers at 0.1 mM each. Reactions conditions were: 94°C 2 min, followed by 25 cycles with 95°C for 30 sec., 55°C for 30 sec and 70°C for 45 sec. Real time PCR with 1–5 µl from the primary PCR and taqman probes were run and analyzed on an ICycler real time PCR machine (Bio-Rad). PCR conditions were done following manufacturer's directions QuantiTect Probe PCR kit (Qiagen): anti-taq antibody inactivation, 95°C (15 min); 40 cycles of 95°C for 30 sec, 62°C for 30 sec and 72°C for 30 sec.

For each RNA sample, the control transcript (*sigA*) and target mRNAs were reverse-transcribed together in one reaction and the resulting cDNAs were quantified by real time PCR. The target cDNA was normalized internally to the *sigA* cDNA levels in the same sample and expressed as (target mRNA)/(*sigA* mRNA) [48]. The resulting number was then divided by the values obtained in the same manner from log phase *in vitro* samples.

### Microarray analysis

Microarray analysis was performed using arrays provided by TIGR under the NIAID contract N01-AI-15447 using protocols publicly available from TIGR [49]. Briefly, three µg of total RNA was used to create cDNA labeled with aminoallyl dUTP (Fermentas). Fluorescent Cy3 and Cy5 dyes (Amersham) were then covalently attached to the aminoallyl tags. Each pair of differentially labeled probes was resuspended in 60 µl of hybridization buffer (500 µl formamide, 250 µl 20X SSC, 5 µl 10% SDS, 245 µl ultrapure water) and hybridized to the microarray slide overnight in a 42°C incubator. Slides were then washed in increasingly stringent wash conditions (5 min 1X SSC 0.1% SDS, 10 min 0.1X SSC 0.1% SDS, 4 times 1 minute in 0.1 X SSC and a final 10 second wash in 0.01X SSC). Arrays were scanned and spots were quantified using Genepix 4000B with GenePix 6.0 software. These data were exported to Acuity 4.1 for lowess normalization and analysis. Spots flagged as ‘Not Found’ by the GenePix algorithms were excluded from downstream analysis. Sets of identical arrays were analyzed using the Statistical Analysis of Microarrays (SAM) freely available from Stanford University [50]. SAM generates a false discovery rate value for each gene. Unless otherwise noted, significance was defined as having a mean log_2_ fold change of 1 with a FDR of <0.4% in a minimum of three arrays.

## Supporting Information

Figure S1 Survival of H37Rv and H37Rv:ΔdosR in hypoxia-induced bacteriostasis in vitro. Colony forming units per ml of parent strain H37Rv (black bar) and H37Rv:ΔdosR (white bar) were counted at each time point in the defined model of hypoxia. Data are from one representative experiment of two. Each bar represents a minimum of 2 biological replicates.
(0.18 MB TIF)

Figure S2 The phenotype of H37RvΔdosR in mouse model is indistinguishable from wild type in C3H/FEJ. Bacterial burdens of H37Rv (squares) and H37RvΔdosR (triangles) in the (A) lungs and (B) spleens of C3H/FEJ mice over the course of infection. Each point represents the average of five experiments with 3–5 animals per time point in each experiment. Error bars represent standard deviation.
(0.23 MB TIF)

Figure S3 The phenotype of H37Rv:ΔdosR in mouse model is indistinguishable from wild type in DBA/2J mice. Bacterial burdens of H37Rv (squares) and H37Rv:ΔdosR (triangles) in the (A) lungs and (B) spleens of C3H/FEJ mice over the course of infection. Mice were inected via tail vein injection with 10^6^ cfu MTB. Each point represents the average of five experiments with 3–5 animals per time point in each experiment. Error bars represent standard deviation.
(0.24 MB TIF)

Table S1 DosR regulon mean expression levels over the hypoxic time course.
(0.18 MB DOC)

Table S2 EHR over the hypoxic time course.
(0.36 MB DOC)
