# Supplementary material for: The Enduring Hypoxic Response of Mycobacterium tuberculosis
Source: PLoS One. 2008 Jan 30;3(1):e1502. doi: 10.1371/journal.pone.0001502 (PMC2198943; doi:10.1371/journal.pone.0001502)
Supplement: Table S1 — DosR regulon mean expression levels over the hypoxic time course. (0.18 MB DOC) [file pone.0001502.s004.doc]

**Table S1. DosR regulon mean expression levels over the hypoxic time course. Values are**

**median log base 2 hypoxia/log phase.**

Values shown are median log base 2 (hypoxia/log phase).
