# Supplementary material for: The Enduring Hypoxic Response of Mycobacterium tuberculosis
Source: PLoS One. 2008 Jan 30;3(1):e1502. doi: 10.1371/journal.pone.0001502 (PMC2198943; doi:10.1371/journal.pone.0001502)
Supplement: Table S2 — EHR over the hypoxic time course. (0.36 MB DOC) [file pone.0001502.s005.doc]

Table S2. EHR over the hypoxic time course.

| **Rv#** | **Gene** | **4 hour** | **8 hour** | **12 hour** | **1 day** | **4 day** | **7 day** |
| --- | --- | --- | --- | --- | --- | --- | --- |
| Rv0031 | Rv0031 | 0.14 | 0.67 | 1.13 | 1.14 | 1.09 | 1.28 |
| Rv0048c | Rv0048c | 0.03 | 1.07 | 1.40 | 1.46 | 1.39 | 2.04 |
| Rv0084 | hycD | 0.67 | 0.31 | 0.33 | 0.69 | 1.03 | 1.45 |
| Rv0089 | Rv0089 | 0.41 | 1.50 | 1.43 | 1.22 | 1.12 | 1.28 |
| Rv0116c | Rv0116c | 0.52 | 1.00 | 0.95 | 1.28 | 1.60 | 1.53 |
| Rv0140 | Rv0140 | 0.12 | 0.74 | 1.37 | 0.96 | 1.87 | 3.18 |
| Rv0171 | mce1C | -0.29 | 0.34 | 0.61 | 0.47 | 1.08 | 1.30 |
| Rv0186 | bglS | 0.76 | 1.08 | 1.51 | 1.26 | 2.09 | 1.04 |
| Rv0188 | Rv0188 | 0.87 | 1.10 | 1.22 | 0.88 | 1.87 | 2.64 |
| Rv0196 | Rv0196 | 0.45 | 1.66 | 1.74 | 1.41 | 1.09 | 1.51 |
| Rv0209 | Rv0209 | 0.37 | 1.05 | 1.25 | 1.00 | 1.38 | 1.03 |
| Rv0232 | Rv0232 | 0.90 | 0.90 | 0.72 | 0.65 | 1.33 | 1.93 |
| Rv0233 | nrdB | 1.56 | 2.69 | 2.52 | 2.36 | 2.99 | 2.49 |
| Rv0244c | fadE5 | 0.66 | 0.26 | 0.18 | 0.44 | 1.58 | 1.50 |
| Rv0250c | Rv0250c | -1.34 | -0.30 | 0.95 | 1.53 | 1.93 | 2.02 |
| Rv0251c | hsp | 0.25 | 0.79 | 2.68 | 3.30 | 3.72 | 4.12 |
| Rv0268c | Rv0268c | 0.61 | 0.65 | 0.78 | 0.79 | 1.54 | 2.80 |
| Rv0324 | Rv0324 | 0.22 | 0.87 | 1.07 | 1.23 | 1.69 | 1.40 |
| Rv0325 | Rv0325 | 0.27 | 0.66 | 1.22 | 1.48 | 1.82 | 1.89 |
| Rv0327c | cyp135A1 | -0.23 | 0.00 | 0.00 | 2.05 | 1.90 | 2.92 |
| Rv0328 | Rv0328 | 0.35 | 1.00 | 1.03 | 0.92 | 1.81 | 1.69 |
| Rv0347 | Rv0347 | 0.13 | 1.21 | 1.42 | 1.47 | 1.34 | 2.11 |
| Rv0349 | Rv0349 | 0.05 | 0.61 | 1.02 | 1.42 | 1.13 | 1.54 |
| Rv0350 | dnaK | 0.42 | 2.20 | 2.38 | 1.88 | 1.69 | 2.33 |
| Rv0352 | dnaJ1 | -0.21 | 0.70 | 0.82 | 0.81 | 1.62 | 1.48 |
| Rv0367c | Rv0367c | 0.01 | -0.35 | 0.18 | 0.93 | 2.35 | 1.20 |
| Rv0384c | clpB | 0.27 | 1.91 | 2.83 | 2.53 | 2.63 | 2.94 |
| Rv0387c | Rv0387c | 0.38 | 0.52 | 0.72 | 0.99 | 1.52 | 1.15 |
| Rv0388c | PPE9 | 0.02 | -0.06 | -0.23 | 0.65 | 1.34 | 1.14 |
| Rv0474 | Rv0474 | 0.71 | 1.09 | 1.46 | 2.25 | 3.97 | 3.39 |
| Rv0494 | Rv0494 | 0.40 | 0.94 | 1.21 | 0.80 | 1.36 | 1.51 |
| Rv0520 | Rv0520 | 0.13 | 0.84 | 1.08 | 1.13 | 1.84 | 3.30 |
| Rv0521 | Rv0521 | 0.23 | 1.00 | 1.40 | 1.29 | 1.52 | 2.32 |
| Rv0551c | fadD8 | -0.28 | 0.99 | 1.15 | 0.77 | 1.22 | 1.18 |
| Rv0563 | htpX | -0.29 | 1.09 | 2.06 | 1.95 | 2.00 | 1.51 |
| Rv0575c | Rv0575c | 0.31 | 0.08 | 0.23 | 0.65 | 1.09 | 1.59 |
| Rv0614 | Rv0614 | 0.20 | 0.99 | 1.18 | 1.17 | 1.37 | 1.83 |
| Rv0653c | Rv0653c | 0.22 | 0.34 | 0.34 | 0.92 | 1.69 | 1.46 |
| Rv0654 | Rv0654 | 0.54 | 0.58 | 0.27 | 0.76 | 1.30 | 1.70 |
| Rv0670 | end | 0.28 | 0.98 | 1.36 | 0.86 | 1.85 | 1.48 |
| Rv0687 | Rv0687 | 0.59 | 1.54 | 2.00 | 1.93 | 2.74 | 2.07 |
| Rv0726c | Rv0726c | 0.29 | 1.61 | 2.12 | 1.98 | 2.67 | 2.03 |
| Rv0753c | mmsA | 0.35 | 1.10 | 1.54 | 1.56 | 1.40 | 1.74 |
| Rv0754 | PE_PGRS11 | 0.75 | 0.67 | 1.57 | 2.73 | 3.05 | 3.06 |
| Rv0757 | phoP | 0.16 | 0.84 | 0.90 | 1.05 | 1.45 | 1.41 |
| Rv0763c | Rv0763c | 0.00 | 1.81 | 2.18 | 1.82 | 2.06 | 1.15 |
| Rv0766c | cyp123 | 0.09 | 0.88 | 1.75 | 2.34 | 3.65 | 3.04 |
| Rv0767c | Rv0767c | 0.17 | 0.45 | 1.33 | 2.67 | 4.72 | 4.02 |
| Rv0791c | Rv0791c | 0.02 | 0.40 | 2.04 | 2.65 | 3.41 | 3.04 |
| Rv0792c | Rv0792c | 0.20 | 0.85 | 1.02 | 1.66 | 1.78 | 1.84 |
| Rv0793 | Rv0793 | 0.14 | 0.27 | 0.81 | 2.13 | 2.71 | 2.73 |
| Rv0826 | Rv0826 | 0.35 | 0.06 | 0.42 | 1.89 | 3.70 | 3.48 |
| Rv0827c | Rv0827c | 1.54 | 1.26 | 1.53 | 1.25 | 1.67 | 1.80 |
| Rv0834c | PE_PGRS14 | 0.64 | 0.80 | 0.72 | 1.03 | 1.34 | 1.59 |
| Rv0846c | Rv0846c | 0.64 | 2.27 | 4.17 | 3.21 | 2.78 | 3.54 |
| Rv0847 | lpqS | 2.17 | 4.93 | 5.50 | 5.35 | 5.04 | 5.56 |
| Rv0872c | PE_PGRS15 | 0.58 | 0.88 | 0.57 | 0.93 | 1.03 | 1.32 |
| Rv0940c | Rv0940c | 0.70 | 1.66 | 1.79 | 1.90 | 3.03 | 2.68 |
| Rv0967 | Rv0967 | 0.32 | 2.59 | 3.99 | 3.84 | 3.99 | 4.81 |
| Rv0976c | Rv0976c | 1.53 | 1.95 | 1.63 | 2.05 | 3.66 | 3.11 |
| Rv0986 | Rv0986 | -0.71 | 0.97 | 1.11 | 1.31 | 1.05 | 2.53 |
| Rv0989c | grcC2 | 0.06 | -0.43 | 0.32 | 1.22 | 1.57 | 1.94 |
| Rv0990c | Rv0990c | 1.71 | 3.97 | 3.80 | 2.60 | 3.18 | 3.90 |
| Rv0991c | Rv0991c | 0.23 | 4.04 | 4.03 | 3.42 | 3.41 | 5.23 |
| Rv1048c | Rv1048c | -0.23 | -0.02 | 1.05 | 1.71 | 3.18 | 2.27 |
| Rv1082 | mca | 0.32 | 0.69 | 1.04 | 1.26 | 1.42 | 1.07 |
| Rv1088 | PE9 | 0.36 | 0.57 | 1.10 | 0.81 | 1.28 | 1.24 |
| Rv1152 | Rv1152 | 0.20 | 0.20 | 0.45 | 1.16 | 1.50 | 1.04 |
| Rv1169c | PE11 | -0.11 | 1.63 | 3.40 | 2.70 | 1.80 | 3.22 |
| Rv1221 | sigE | -0.66 | 1.11 | 1.61 | 1.28 | 2.09 | 2.77 |
| Rv1243c | PE_PGRS23 | 0.26 | 0.17 | 0.20 | 0.39 | 1.01 | 1.00 |
| Rv1255c | Rv1255c | -0.58 | -0.23 | -0.45 | 0.07 | 2.19 | 1.51 |
| Rv1256c | cyp130 | -0.26 | -0.17 | 0.20 | 0.71 | 2.13 | 1.48 |
| Rv1284 | Rv1284 | 0.49 | 1.65 | 2.12 | 1.85 | 3.02 | 3.11 |
| Rv1375 | Rv1375 | -0.03 | 0.33 | 0.66 | 0.74 | 1.00 | 1.15 |
| Rv1403c | Rv1403c | 0.40 | 0.74 | 1.69 | 2.56 | 3.43 | 3.98 |
| Rv1405c | Rv1405c | 1.03 | 2.13 | 2.87 | 2.54 | 3.03 | 3.37 |
| Rv1470 | trxA | 0.22 | 0.47 | 1.26 | 1.11 | 1.29 | 1.91 |
| Rv1471 | trxB1 | -0.17 | 1.62 | 2.66 | 2.85 | 3.10 | 4.56 |
| Rv1472 | echA12 | 0.04 | -0.07 | 0.04 | 0.62 | 1.08 | 1.93 |
| Rv1578c | Rv1578c | 1.02 | 0.51 | 1.28 | 1.32 | 2.10 | 1.58 |
| Rv1623c | cydA | -0.12 | 0.72 | 1.06 | 0.74 | 1.27 | 1.28 |
| Rv1628c | Rv1628c | 0.85 | 1.65 | 1.55 | 1.26 | 1.26 | 1.27 |
| Rv1652 | argC | 0.92 | 0.59 | 1.14 | 1.54 | 3.18 | 1.15 |
| Rv1669 | Rv1669 | 0.00 | 0.04 | 0.29 | 0.53 | 1.18 | 1.16 |
| Rv1733c | Rv1733c | 2.27 | 2.05 | 2.84 | 1.75 | 1.64 | 2.30 |
| Rv1744c | Rv1744c | 0.11 | 1.04 | 1.33 | 0.87 | 1.25 | 1.53 |
| Rv1755c | plcD | 0.11 | 1.52 | 1.76 | 1.16 | 1.13 | 1.31 |
| Rv1760 | Rv1760 | -0.07 | 1.16 | 1.61 | 1.99 | 1.77 | 2.30 |
| Rv1766 | Rv1766 | -0.27 | 0.10 | 0.25 | 0.45 | 1.54 | 2.17 |
| Rv1806 | PE20 | 0.35 | 0.20 | 0.70 | 0.85 | 1.40 | 4.39 |
| Rv1874 | Rv1874 | -0.28 | -0.20 | 0.04 | 0.12 | 1.04 | 3.39 |
| Rv1875 | Rv1875 | 0.16 | 0.25 | 0.53 | 0.88 | 1.95 | 3.75 |
| Rv1888c | Rv1888c | 0.84 | 0.76 | 1.37 | 1.05 | 1.82 | 2.31 |
| Rv1891 | Rv1891 | 0.27 | 0.26 | 0.54 | 1.07 | 1.44 | 1.08 |
| Rv1893 | Rv1893 | 0.76 | 1.53 | 1.65 | 1.51 | 1.52 | 1.16 |
| Rv1895 | Rv1895 | 0.21 | 0.68 | 0.98 | 1.15 | 1.38 | 1.65 |
| Rv1909c | furA | 0.09 | 1.41 | 1.83 | 1.21 | 1.59 | 2.99 |
| Rv1926c | mpt63 | 0.18 | 0.46 | 1.08 | 0.64 | 1.02 | 1.31 |
| Rv1928c | Rv1928c | 0.16 | -0.13 | 0.11 | 1.17 | 1.23 | 2.27 |
| Rv1951c | Rv1951c | 0.46 | 0.48 | 0.20 | 1.02 | 1.57 | 1.66 |
| Rv1954c | Rv1954c | 0.51 | 0.09 | 0.81 | 1.39 | 2.87 | 2.68 |
| Rv1955 | Rv1955 | 0.19 | 1.26 | 2.01 | 2.51 | 4.30 | 4.38 |
| Rv1956 | Rv1956 | 0.36 | 1.34 | 1.59 | 2.23 | 2.73 | 3.12 |
| Rv1957 | Rv1957 | -0.15 | 0.29 | 0.87 | 1.87 | 3.83 | 3.54 |
| Rv1958c | Rv1958c | -0.04 | 0.44 | 0.62 | 1.55 | 2.22 | 1.88 |
| Rv1959c | Rv1959c | 0.00 | 0.88 | 1.19 | 1.19 | 2.16 | 2.09 |
| Rv1960c | Rv1960c | 0.02 | 0.98 | 1.17 | 0.98 | 1.79 | 1.92 |
| Rv1963c | mce3R | -0.11 | 0.38 | 0.70 | 0.88 | 1.51 | 1.34 |
| Rv1974 | Rv1974 | 0.18 | 1.31 | 1.50 | 1.45 | 1.20 | 1.49 |
| Rv1985c | Rv1985c | 0.07 | 1.32 | 1.93 | 1.84 | 2.44 | 2.10 |
| Rv1989c | Rv1989c | 0.82 | 2.76 | 2.26 | 1.31 | 1.96 | 2.19 |
| Rv1990c | Rv1990c | 0.66 | 2.77 | 2.76 | 1.69 | 2.43 | 3.15 |
| Rv1994c | Rv1994c | 1.21 | 3.80 | 3.68 | 2.84 | 2.22 | 3.22 |
| Rv2011c | Rv2011c | 0.12 | 1.41 | 2.03 | 1.74 | 1.63 | 2.91 |
| Rv2012 | Rv2012 | 0.49 | 0.86 | 1.70 | 1.89 | 3.22 | 3.32 |
| Rv2020c | Rv2020c | -0.15 | -0.08 | 0.17 | 0.72 | 1.15 | 1.68 |
| Rv2021c | Rv2021c | -0.31 | -0.18 | 0.31 | 1.15 | 2.03 | 2.48 |
| Rv2022c | Rv2022c | -0.05 | -0.05 | 0.44 | 0.97 | 2.10 | 2.95 |
| Rv2025c | Rv2025c | 0.84 | 1.89 | 1.29 | 2.10 | 3.04 | 2.88 |
| Rv2034 | Rv2034 | 0.68 | 1.78 | 2.63 | 3.74 | 5.05 | 5.00 |
| Rv2035 | Rv2035 | 0.27 | 1.71 | 2.38 | 3.09 | 3.79 | 3.83 |
| Rv2036 | Rv2036 | 0.73 | 1.50 | 2.24 | 2.59 | 2.04 | 3.31 |
| Rv2050 | Rv2050 | 0.79 | 1.70 | 2.12 | 2.33 | 2.79 | 3.24 |
| Rv2098c | PE_PGRS36 | 0.80 | 0.78 | 1.30 | 1.24 | 1.86 | 1.64 |
| Rv2122c | hisE | 0.19 | 0.46 | 0.42 | 0.82 | 1.43 | 1.26 |
| Rv2123 | PPE37 | 0.52 | 1.56 | 1.70 | 1.22 | 1.43 | 1.26 |
| Rv2137c | Rv2137c | 0.38 | 0.48 | 0.18 | 0.46 | 1.62 | 1.82 |
| Rv2185c | TB16.3 | 0.40 | 0.39 | 0.57 | 0.51 | 1.12 | 1.31 |
| Rv2217 | lipB | 0.34 | 0.23 | 0.27 | 0.32 | 1.02 | 1.38 |
| Rv2243 | fabD | 2.12 | 2.15 | 1.53 | 0.99 | 1.92 | 2.25 |
| Rv2299c | htpG | -0.04 | 0.84 | 1.27 | 1.16 | 1.28 | 1.46 |
| Rv2324 | Rv2324 | -0.04 | 1.00 | 1.31 | 1.17 | 2.18 | 1.67 |
| Rv2359 | furB | 0.94 | 0.88 | 0.82 | 0.88 | 1.50 | 2.20 |
| Rv2374c | hrcA | 0.60 | 1.13 | 1.15 | 0.98 | 1.58 | 1.23 |
| Rv2389c | rpfD | -0.21 | -0.26 | 0.28 | 0.68 | 1.59 | 1.75 |
| Rv2390c | Rv2390c | -0.07 | 0.34 | 0.14 | 0.23 | 1.08 | 1.33 |
| Rv2412 | rpsT | 1.42 | 0.83 | 0.83 | 1.28 | 1.75 | 1.65 |
| Rv2465c | Rv2465c | -0.35 | -0.14 | 0.34 | 0.89 | 1.52 | 2.99 |
| Rv2466c | Rv2466c | 0.05 | 1.65 | 2.47 | 2.93 | 3.05 | 4.15 |
| Rv2497c | pdhA | 0.87 | 1.48 | 1.41 | 1.24 | 1.83 | 1.08 |
| Rv2498c | citE | 0.43 | 1.81 | 1.83 | 1.37 | 1.62 | 1.04 |
| Rv2499c | Rv2499c | 0.51 | 2.00 | 2.14 | 1.62 | 1.73 | 1.08 |
| Rv2500c | fadE19 | 0.29 | 1.79 | 2.22 | 1.21 | 1.34 | 1.38 |
| Rv2504c | scoA | 0.81 | 1.16 | 1.22 | 1.12 | 1.43 | 2.67 |
| Rv2517c | Rv2517c | 0.39 | 2.00 | 2.19 | 1.66 | 2.59 | 2.63 |
| Rv2526 | Rv2526 | 0.05 | 0.29 | 0.43 | 0.71 | 1.20 | 1.04 |
| Rv2527 | Rv2527 | -0.03 | -0.02 | 0.36 | 1.00 | 1.21 | 1.09 |
| Rv2557 | Rv2557 | 0.88 | 1.34 | 1.30 | 0.72 | 1.72 | 2.16 |
| Rv2558 | Rv2558 | 1.50 | 2.49 | 2.23 | 1.17 | 3.10 | 3.64 |
| Rv2583c | relA | 0.94 | 0.35 | 0.28 | 0.30 | 1.21 | 1.10 |
| Rv2617c | Rv2617c | 0.07 | 0.47 | 0.29 | 0.40 | 1.36 | 2.48 |
| Rv2626c | Rv2626c | 3.89 | 5.32 | 5.03 | 4.35 | 4.13 | 4.76 |
| Rv2627c | Rv2627c | 2.32 | 4.43 | 3.34 | 2.63 | 2.52 | 3.28 |
| Rv2628 | Rv2628 | 2.31 | 2.96 | 2.68 | 1.85 | 1.69 | 2.43 |
| Rv2642 | Rv2642 | 0.03 | 0.91 | 2.17 | 2.63 | 4.11 | 4.74 |
| Rv2643 | arsC | 0.37 | 0.36 | 1.44 | 2.26 | 2.83 | 3.73 |
| Rv2653c | Rv2653c | 0.22 | 0.85 | 1.73 | 1.30 | 1.44 | 1.23 |
| Rv2657c | Rv2657c | 0.94 | 1.77 | 1.50 | 0.94 | 1.50 | 1.73 |
| Rv2658c | Rv2658c | 2.15 | 6.08 | 6.01 | 4.68 | 4.93 | 4.29 |
| Rv2660c | Rv2660c | 1.10 | 1.00 | 2.57 | 2.50 | 2.96 | 2.47 |
| Rv2662 | Rv2662 | 0.66 | 2.12 | 1.49 | 1.68 | 1.37 | 3.35 |
| Rv2663 | Rv2663 | 0.44 | 1.44 | 1.37 | 1.30 | 1.24 | 2.90 |
| Rv2664 | Rv2664 | 0.36 | 0.51 | 1.20 | 1.28 | 2.12 | 2.76 |
| Rv2687c | Rv2687c | 0.84 | 1.21 | 1.58 | 1.62 | 1.83 | 1.61 |
| Rv2688c | Rv2688c | 0.71 | 1.59 | 1.81 | 1.84 | 2.49 | 2.21 |
| Rv2693c | Rv2693c | 0.18 | 0.47 | 0.97 | 0.77 | 2.24 | 2.35 |
| Rv2694c | Rv2694c | 0.15 | 1.21 | 1.82 | 1.59 | 2.50 | 2.62 |
| Rv2699c | Rv2699c | -0.49 | 0.30 | 0.74 | 0.57 | 1.21 | 2.63 |
| Rv2700 | Rv2700 | 0.39 | 0.49 | 0.36 | 0.63 | 1.14 | 1.09 |
| Rv2731 | Rv2731 | 0.29 | 0.32 | 0.68 | 0.52 | 1.59 | 1.51 |
| Rv2744c | 35kd_ag | -0.78 | 0.40 | 1.48 | 1.16 | 1.62 | 1.51 |
| Rv2745c | Rv2745c | -1.07 | 1.39 | 2.46 | 2.19 | 2.29 | 2.21 |
| Rv2764c | thyA | 0.37 | 0.53 | 0.78 | 0.83 | 1.22 | 1.49 |
| Rv2830c | Rv2830c | 1.04 | 1.75 | 1.50 | 0.71 | 1.18 | 1.18 |
| Rv2865 | Rv2865 | -0.28 | -0.06 | 0.69 | 0.96 | 1.26 | 1.06 |
| Rv2866 | Rv2866 | -0.20 | 0.41 | 1.01 | 1.00 | 1.67 | 1.13 |
| Rv2876 | Rv2876 | 0.16 | 0.65 | 0.82 | 0.84 | 1.13 | 1.03 |
| Rv2913c | Rv2913c | 0.12 | 0.01 | 0.35 | 0.96 | 3.29 | 3.54 |
| Rv2964 | purU | 0.24 | 1.53 | 1.89 | 2.86 | 2.97 | 1.78 |
| Rv3007c | Rv3007c | -0.10 | 1.03 | 1.41 | 1.15 | 1.31 | 1.49 |
| Rv3016 | lpqA | -0.04 | -0.21 | -0.10 | 0.27 | 1.29 | 1.60 |
| Rv3031 | Rv3031 | 0.01 | 0.47 | 1.00 | 0.63 | 1.01 | 1.04 |
| Rv3066 | Rv3066 | 0.75 | 0.81 | 1.25 | 1.09 | 1.78 | 1.14 |
| Rv3071 | Rv3071 | 0.12 | 1.02 | 1.33 | 1.26 | 1.28 | 1.49 |
| Rv3161c | Rv3161c | -0.01 | 0.60 | 0.93 | 0.90 | 1.51 | 1.42 |
| Rv3174 | Rv3174 | 0.35 | 0.19 | -0.09 | 0.42 | 1.04 | 1.68 |
| Rv3175 | Rv3175 | 0.23 | 0.04 | 0.64 | 0.11 | 1.57 | 1.75 |
| Rv3176c | mesT | 0.66 | 0.61 | 1.15 | 1.04 | 2.95 | 1.86 |
| Rv3180c | Rv3180c | 0.24 | 0.06 | 0.10 | 0.59 | 1.13 | 1.23 |
| Rv3182 | Rv3182 | 0.72 | 1.81 | 1.88 | 1.25 | 1.23 | 1.63 |
| Rv3188 | Rv3188 | 0.73 | 0.16 | 0.03 | 1.00 | 1.46 | 1.61 |
| Rv3197A | whiB7 | 0.13 | 0.75 | 1.12 | 0.46 | 1.21 | 1.72 |
| Rv3206c | moeB1 | 0.05 | 0.30 | 0.56 | 0.92 | 1.27 | 1.95 |
| Rv3221A | Rv3221A | 0.04 | 0.40 | 0.82 | 0.94 | 1.64 | 2.27 |
| Rv3223c | sigH | 0.51 | -0.49 | -0.21 | 0.62 | 1.01 | 1.78 |
| Rv3269 | Rv3269 | 2.79 | 2.30 | 2.39 | 2.14 | 3.17 | 2.93 |
| Rv3290c | lat | 0.79 | 5.08 | 4.42 | 2.64 | 2.10 | 3.22 |
| Rv3334 | Rv3334 | 0.19 | 2.17 | 2.95 | 2.63 | 2.45 | 3.40 |
| Rv3343c | PPE54 | 0.31 | -0.20 | -0.11 | 1.11 | 1.11 | 1.63 |
| Rv3406 | Rv3406 | 0.25 | -0.50 | -0.12 | 0.71 | 2.85 | 4.31 |
| Rv3418c | groES | -2.14 | 1.84 | 1.97 | 1.16 | 1.53 | 1.95 |
| Rv3480c | Rv3480c | 0.46 | 0.64 | 1.18 | 1.26 | 2.28 | 1.69 |
| Rv3503c | fdxD | 1.22 | 1.73 | 1.83 | 1.72 | 2.96 | 2.55 |
| Rv3508 | PE_PGRS54 | 0.33 | 0.80 | 0.88 | 0.86 | 1.16 | 1.35 |
| Rv3515c | fadD19 | 0.31 | 1.67 | 1.90 | 2.87 | 3.02 | 2.99 |
| Rv3526 | Rv3526 | 0.50 | 1.73 | 1.75 | 2.03 | 1.82 | 2.05 |
| Rv3527 | Rv3527 | 0.46 | 1.58 | 1.51 | 1.52 | 1.86 | 1.86 |
| Rv3530c | Rv3530c | 0.77 | 0.55 | 2.00 | 1.98 | 2.10 | 1.78 |
| Rv3531c | Rv3531c | 0.49 | 0.41 | 1.32 | 1.94 | 2.05 | 2.02 |
| Rv3533c | PPE62 | -0.20 | 0.22 | 0.38 | 1.64 | 1.22 | 2.12 |
| Rv3534c | Rv3534c | 1.13 | 2.74 | 3.03 | 2.85 | 3.90 | 1.72 |
| Rv3536c | Rv3536c | 0.46 | 0.94 | 1.30 | 1.55 | 3.34 | 3.89 |
| Rv3537 | Rv3537 | 1.43 | 1.34 | 2.08 | 2.44 | 2.65 | 1.49 |
| Rv3545c | cyp125 | 0.73 | 1.06 | 1.15 | 1.11 | 1.48 | 1.12 |
| Rv3571 | hmp | 0.33 | 1.28 | 1.18 | 1.68 | 2.05 | 1.95 |
| Rv3572 | Rv3572 | 0.08 | 0.91 | 1.00 | 1.25 | 1.71 | 1.20 |
| Rv3574 | Rv3574 | 0.59 | 1.26 | 2.15 | 1.70 | 2.52 | 1.63 |
| Rv3597c | lsr2 | -0.02 | -0.15 | 0.07 | 0.35 | 2.14 | 2.35 |
| Rv3628 | ppa | 0.77 | 0.73 | 0.87 | 1.15 | 1.54 | 1.49 |
| Rv3681c | whiB4 | 0.99 | 1.65 | 1.75 | 1.39 | 1.81 | 2.62 |
| Rv3747 | Rv3747 | -0.21 | 0.08 | 0.22 | 0.95 | 1.23 | 1.61 |
| Rv3819 | Rv3819 | -0.03 | 0.59 | 0.74 | 0.90 | 1.01 | 1.16 |
| Rv3832c | Rv3832c | 0.11 | 0.52 | 0.70 | 1.02 | 1.29 | 1.47 |
| Rv3837c | Rv3837c | 0.34 | 1.10 | 1.40 | 1.32 | 1.44 | 1.73 |
| Rv3848 | Rv3848 | 0.32 | 0.55 | 0.87 | 1.03 | 1.37 | 1.62 |
| Rv3862c | whiB6 | 0.62 | 0.60 | 1.55 | 2.32 | 3.98 | 4.30 |
| Rv3864 | Rv3864 | 0.00 | 1.02 | 1.64 | 1.15 | 1.44 | 1.04 |
| Rv3903c | Rv3903c | -0.09 | 0.08 | 0.31 | 0.88 | 1.01 | 1.61 |
| Rv3913 | trxB2 | 0.24 | 0.75 | 0.99 | 0.99 | 1.43 | 1.57 |

Values shown are median log base 2 (hypoxia/log phase).
